# Attributable Risk of Hypertension for Cardiovascular Disease

**DOI:** 10.1007/s11906-026-01363-w

**Published:** 2026-03-26

**Authors:** Hunter P. Mace, Donald Clark

**Affiliations:** 1 Department of Internal Medicine, University of Alabama at Birmingham, Birmingham, AL US; 2 Department of Medicine, Division of Cardiology, University of Mississippi Medical Center, Jackson, MS US

**Keywords:** Hypertension, Blood pressure

## Abstract

**Purpose of Review:**

Cardiovascular disease (CVD) remains the leading cause of mortality worldwide, and hypertension is a modifiable risk factor with the strongest association for developing heart disease. This review evaluates the attributable risk of hypertension for CVD at the population level and examines how this risk varies by demographic, geographic, and socioeconomic factors. Understanding the extent to which hypertension contributes to CVD can help inform targeted prevention and intervention treatment strategies.

**Recent Findings:**

Recent studies have examined the attributable risk of hypertension for CVD across different populations. Hypertension had the highest population attributable risk (PAR) and population attributable fraction (PAF) values for overall CVD, stroke, heart failure, and coronary heart disease, especially among Black adults, younger individuals, and residents of low- and middle-income countries. Although hypertension prevalence has increased over time, some high-income countries have observed a decline in its attributable risk for CVD. This decline is likely due to improved blood pressure management and public health interventions. In the US, recent data shows blood pressure control among adults remains low. Population-level interventions such as sodium restrictions in the food supply, standardized treatment protocols to improve blood pressure control, and improved healthcare access have been shown to mitigate the burden of hypertension.

**Summary:**

Despite advancements in awareness and treatment, hypertension continues to be a major driver of the global CVD burden. The meaningful attributable risk for CVD highlights the need for improved public health protocols. Interventions that combine clinical management with population-level prevention strategies offer the greatest potential for reducing disparities and improving cardiovascular outcomes.

## Introduction

Hypertension is one of the most prevalent non-communicable medical conditions worldwide and is a significant public health concern due to its strong association with cardiovascular disease (CVD) [1]. CVD, including coronary heart disease (CHD), stroke, and heart failure (HF), remains the leading cause of mortality worldwide, and the burden continues to rise with a growing and aging population [2]. In the United States, 47% of the population, or 122.4 million adults are estimated to be affected by high blood pressure [3]. The substantial prevalence of hypertension and the widespread availability of effective medication treatments that reduce the risk for CVD highlights the importance of effective public health strategies to increase awareness and blood pressure control. There are many other modifiable risk factors for CVD such as cigarette smoking, hyperlipidemia, diabetes, obesity, and physical inactivity, and studies have been conducted to estimate the attributable risk of certain risk factors to developing CVD.

Calculations such as the population-attributable risk (PAR) and population-attributable fraction (PAF) are important epidemiological tools used to quantify the proportion of disease incidence in a population that can be attributed to a specific risk factor [4, 5]. In diseases with many exposures, the PAR and the PAF can estimate the theoretical effect that removing a specific risk factor would have on the overall disease burden. Both measures account for the prevalence of a risk factor in its calculation, thus risk factors with greater prevalence will have a higher PAR or PAF. The key difference between these values is the calculation of an absolute difference in disease incidence (PAR) versus a relative difference in disease incidence displayed as a percentage of the disease attributable to the exposure (PAF).

Interpretation of PAR and PAF is limited by the assumption that risk factors act independently. In practice, cardiovascular risk factors frequently co-occur and are biologically interrelated—for example, obesity and hypertension often cluster—resulting in overlapping attributable risk. Consequently, PAR and PAF estimates for individual exposures cannot be summed without overestimating the preventable disease burden. Despite these limitations, understanding the attributable risk of hypertension for CVD is vital for public health policy as it can guide interventions and resource allocation that can have the greatest impact on reducing the burden of CVD. This review analyzes the current literature on the attributable risk of hypertension as a modifiable risk factor for the development of cardiovascular disease.

## Epidemiology of Hypertension

Hypertension is highly prevalent globally in both developed and developing nations and is estimated to affect nearly one-third of the world’s adult population [6]. According to the 2017 American College of Cardiology/American Heart Association (ACC/AHA) blood pressure guidelines, hypertension is defined as a systolic blood pressure ≥ 130 mmHg, diastolic blood pressure ≥ 80 mmHg, or antihypertensive medication use [7]. Of the estimated 122.4 million adults in the United States with hypertension, 51.3% are male and 48.7% are female [3]. In the National Health and Nutrition Examination Survey (NHANES) 2017 to 2020, the prevalence of hypertension increased with age. The prevalence of hypertension was 28.5% among adults 20–44 years of age and rose to 76.5% among those ≥ 65 years of age [3]. Hypertension disproportionately affects Black adults in the United States with a prevalence of 55.8% among Black males and 56.9% among Black females compared to a prevalence of 46.6% among White males and 40% among White females [3]. There is also a geographic disparity among states with the highest levels of hypertension in the Southeastern states. Mississippi ranks highest with an estimated hypertension prevalence of 42.4%, while Utah is the lowest with an estimated prevalence of 23.7%[8].

Studies have also shown that social determinants of health can have an impact on the prevalence of hypertension. A meta-analysis of 51 studies showed an overall increased risk of hypertension among those with the lowest socioeconomic status (SES) using income, occupation, and education as indicators of SES [9]. An analysis of the Jackson Heart Study cohort focused on the effect of SES on hypertension prevalence and incidence in the Black population. Factors such as high adult income, occupation, and wealth were inversely associated with hypertension prevalence and higher childhood SES was associated with reduced prevalent and incident hypertension [10]. 

## Attributable Risk of Hypertension for CVD

Given the high prevalence and strong association with CVD, high blood pressure is an important risk factor that can account for a significant portion of cardiovascular disease [11]. As shown in Table 1, several studies have been conducted to quantify the attributable risk of hypertension for CVD in different patient populations around the world. These studies have used the prevalence of hypertension in select cohorts with the incidence of CVD to quantify the attributable risk of hypertension, as well as other risk factors associated with the development of CVD.

**Table 1:** The population attributable risk/population attributable fraction of hypertension for overall CVD across studies

Study Title	PAR/PAF
Clark D III et al. (JAMA Cardiol. 2019)	32.50% (PAR)
Willey JZ et al. (J Am Heart Assoc. 2014)	24.30% (PAR)
Luo D et al. (BMJ. 2020)	23.80% (PAF)
Tian F et al. (EClinicalMedicine. 2023)	14.04% (PAF)
Cheng S et al. (Circulation. 2014)	25.00% (PAR)
Yusuf S et al. (Lancet. 2020)	22.30% (PAF)
Li S et al. (Eur Heart J. 2022)	25.00% (PAF)

### Race, Sex, and Age Differences

There are important and well-established racial disparities for the prevalence of hypertension and CVD. An analysis of the Atherosclerosis Risk in Communities (ARIC) Study compared CVD risk factors in Black and White adults and found that the proportion with all optimal risk factors was lower in Black adults (3.8%) compared to White adults (7.5%)[12]. Additionally, Black adults were more likely to have at least 1 elevated risk factor compared to White adults (80% versus 60%). After adjustment for risk factor differences and education level, the proportion of CVD events explained by elevated risk factors was 90% in Black adults compared with only 65% in White adults. These findings highlight the importance of targeting CVD risk factors to address racial disparities in CVD.

A cohort study utilizing data from the Jackson Heart Study, REGARDS study, and NHANES was performed to examine the population-attributable risk for CVD associated with hypertension among Black adults [13]. The prevalence of hypertension was 53.2% among non-Hispanic Black adults, and the PAR associated with hypertension for overall CVD in this population was 32.5%. By sex, the PAR for women was 31.1% and 33.9% for men. When analyzed for specific CVDs, hypertension had the greatest attributable risk for CHD with a PAR of 42.7%. Stroke and heart failure were also significantly associated with PARs of 38.9% and 21.6%, respectively. Importantly, a PAR of 69% for stroke associated with hypertension was present among those younger than 60 years old. This finding correlates with a previous study that found the PAR of hypertension for stroke was greater in adults under the age of 80 years old (35.6%) compared to those greater than 80 years old (−0.3%)[15].

Other cohort studies have also shown racial differences for the PAR associated with HTN for stroke and HF[14, 15]. A pooled cohort of White and Black adults in the United States found that PAF for HF associated with hypertension was highest among all CVD risk factors in Black men and women, 28.3% and 25.8%, respectively [14]. In White adults, the PAF for HF associated with hypertension was 17.3% in men and women. A similar cohort study of a multiethnic elderly population showed a substantial gap in the PAR associated with hypertension for stroke in Hispanic adults (50.6%) and Black adults (16%) compared with White adults (2.6%)[15]. Hypertension is more prevalent in the Black population, and the rates of blood pressure control are lower than in other populations and have been decreasing over time [16, 17]. Overall, these findings suggest that tailored prevention strategies focused on hypertension control in minority populations may have the highest impact to mitigate racial disparities in CVD burden.

**Fig. 1:**
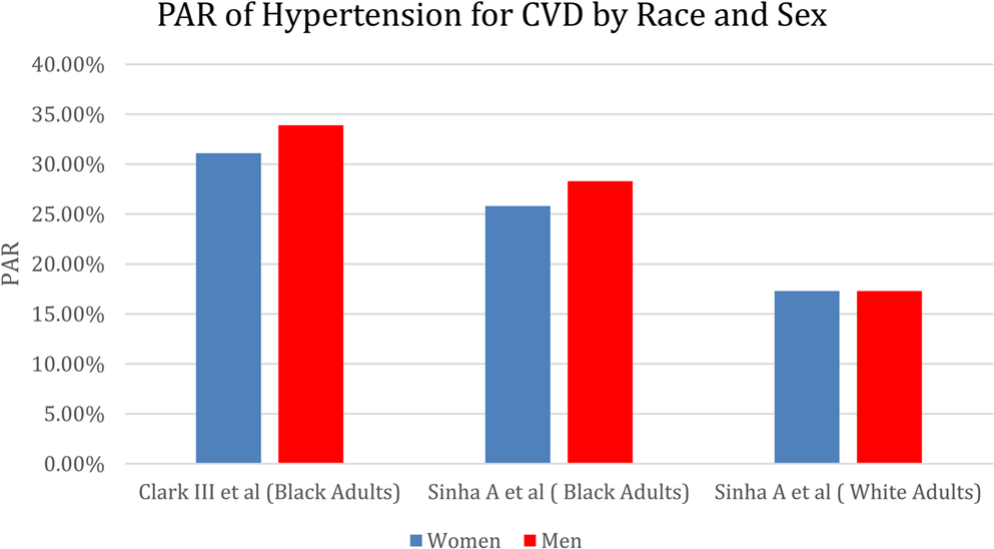
The Population Attributable Risk of Hypertension for Cardiovascular Disease by Race and Sex across Studies

The attributable risk of hypertension for CVD can be significant across age groups and has been evaluated in several studies. Hypertension among young adults is a growing problem as prevalence and related CVD deaths have risen over time [18]. A systemic review and meta-analysis was performed to investigate adverse outcomes in young adults aged 18–45 with hypertension [19]. This study found the PAF for CVD associated with hypertension was 23.8% among this cohort of young adults, with a graded, progressive association of blood pressure with increased risk of adverse CVD events [19]. This high attributable risk for CVD associated with high blood pressure highlights the significant effect hypertension can have on young adults and the need for widespread screening and treatment. An additional study examined the age-specific variations in modifiable risk factors for CVD among middle-aged adults (< 50 years), quinquagenarians (50 to < 60 years), and the elderly (> 60 years) [20]. The PAF for hypertension increased with age and was the most significant risk factor for CVD among quinquagenarians and the elderly. Collectively, these findings demonstrate the substantial impact on CVD burden as the prevalence of elevated BP and hypertension increases across age spectrums. Figure 1.

### Temporal Trends

An analysis of the ARIC Study provided insight into the changing contribution of risk factors for the development of CVD over time from 1987 to 1998 [21]. Hypertension was the greatest and most consistent risk factor for population CVD risk over this time frame. The prevalence of hypertension in the cohort increased from 39% to 45%, and the PAR for CVD associated with hypertension in the total sample stayed consistent at 25%[21]. While the cohort showed a stable overall PAR over time, significant differences were noted when analyzed by sex and race. The PAR of hypertension for CVD decreased from 23% to 19% in men and increased from 28% to 32% in women despite the prevalence of hypertension increasing in both sexes. Important racial differences in the temporal trends of PAR for CVD were also observed. The PAR of hypertension for CVD in the Black cohort decreased over time from 40% to 36% but was substantially higher than the PAR in the White cohort which was stable at 21%. The risk attributable to hypertension remains high in women as well as Black adults.

An analysis of the Rotterdam Study evaluated temporal trends in PAFs of modifiable risk factors for CVD across three decades from 1989 to 2014 [22]. The cohort was divided into three time groups of individuals without CVD with a mean age of 70 years and followed for five years. The prevalence of hypertension and other individual risk factors such as obesity and diabetes rose over time in both sexes, however, the attributable risk of CVD associated with hypertension decreased over time. Hypertension was the single largest contributor to overall CVD in the 1990 s (37%) and 2000 s (34%), but later significantly decreased in the 2010 s (12%) in all populations. A similar decrease was noted in both sexes. This study also investigated the PAF of risk factors for specific CVDs such as coronary heart disease, stroke, and heart failure. The PAF of hypertension for these three diseases was high in the 1990 s and 2000 s but decreased substantially and was not statistically significant in the 2010s. The fact that hypertension has increased in prevalence, but decreased in attributable risk to CVD likely indicates improvement in public health management in the Netherlands and could serve as a guide to management in other populations.

### Across Economic Levels

A multinational cohort study examined the associations of multiple CVD risk factors across 21 countries divided in groups by high-income (HIC), middle-income (MIC), and low-income (LIC) countries [23]. Behavioral, metabolic, socioeconomic, and psychosocial risk factors were assessed. Overall, 70% of CVD was attributable to individual and household risk factors. Metabolic risk factors made up the largest contribution across all groups of countries by income, and hypertension was the greatest single contributor with a PAF of 22.3% for CVD. Hypertension was also a greater risk factor for stroke than myocardial infarction. When stratified by income, hypertension was the number one attributable risk factor for developing CVD in MIC (PAF 26.5%) and LIC (PAF 14.3%), however, it ranked third behind high non-HDL cholesterol (PAF 20.7%) and tobacco use (PAF 15.7%) in HIC (PAF 14.6%).

Other studies have shown similar levels of attributable risk of hypertension in developing nations, demonstrating hypertension to be the greatest contributor to CVD death [24]. Across the income-categorized countries, hypertension had the greatest PAF for death in MIC (PAF 13.2%)[23]. A similar analysis of the PURE prospective cohort study found the greatest incidence of CVD among men compared to women and in rural areas compared to urban areas [25]. Hypertension had the greatest PAF among risk factors for CVD incidence (25.0%) and for death (10.8%). Hypertension in the setting of other socioeconomic risk factors such as low income, low education, and poor diet can lead to increased incidence of CVD and worse outcomes.

## Public Health Approaches to Hypertension Management

Hypertension represents a significant public health concern given its high prevalence and significant risk for developing CVD. Models have estimated that every 10% increase in hypertension treatment would lead to the prevention of 14,000 deaths per year in those aged less than 80 years old [26]. While general knowledge, awareness, and treatment of hypertension have improved over time, there continues to be a lack a comprehensive understanding of the condition and recent evidence demonstrates an alarming decline in control rates [27, 28]. In a comprehensive analysis of NHANES data, prevalence of controlled BP increased between 1999 and 2000 and 2007–2008, did not significantly change from 2007 to 2008 through 2013–2014, and then decreased after 2013-2014 [29]. These findings were accompanied by the Surgeon General’s Call to Action to Control Hypertension to make hypertension control a national priority in the United States, ensure that communities support hypertension control, and optimize patient care for hypertension control [30]. 

While individual-based management of BP control is important, broader population-level interventions could be beneficial to improve the management of the hypertension epidemic [31–35]. Success in reducing the burden of other health problems that require long-term treatment has shown that large-scale, standardized protocols can improve care and patient adherence [35, 36]. The Kaiser Permanente Northern California (KPNC) hypertension program increased blood pressure control from 43.6% to 80.4% over an eight-year duration (2001–2009). In contrast, national mean control improved from 55.4% to 64.1% during this same period [35]. The KPNC program focused on creating a system-wide hypertension registry, developing evidence-based practice guidelines, implementing a follow-up schedule to monitor blood pressure changes, and promoting single-pill combination therapy. The components of this protocol empowered clinicians with effective algorithm-based guidelines to reduce clinical variability and encourage patient adherence.

Improving access to healthy foods and reducing sodium intake could also have a significant impact on hypertension management, and national initiatives are underway to address this issue [37]. Excess sodium intake is associated with hypertension and CVD, and in 2021 the US FDA issued guidance for the voluntary reduction of sodium content in the US food supply to decrease excess sodium consumption in the US[38]. Thus far, the results have been encouraging as preliminary data from 2022 showed 40% of Phase I targets were achieved. In August 2024, the FDA released Phase II of their voluntary sodium reduction recommendations with a 3-year goal of continuing to reduce sodium in the food supply with a goal of average sodium intake of 2750 milligrams per day [39]. This would reflect a 20% decrease from the consumer intake levels before 2021. Even a gradual reduction in the sodium levels of the national food supply could have significant ramifications for the prevention of CVD at the population level [40]. 

## Conclusion

Hypertension remains a substantial risk factor for CVD, contributing to a significant portion of the global cardiovascular disease burden. Despite advances in treatment and management, the prevalence of hypertension remains significantly high and the attributable risk for CVD underscores the need for continued efforts to improve blood pressure control at the population level. Multifaceted public health strategies, including education campaigns, standardized treatment protocols, and sodium reduction in the food supply are essential in reducing the impact of hypertension on adverse cardiovascular outcomes.

## Data Availability

No datasets were generated or analysed during the current study.
